# *Ny*-*1* and *Ny*-*2* genes conferring hypersensitive response to potato virus Y (PVY) in cultivated potatoes: mapping and marker-assisted selection validation for PVY resistance in potato breeding

**DOI:** 10.1007/s11032-014-0024-4

**Published:** 2014-01-23

**Authors:** Katarzyna Szajko, Danuta Strzelczyk-Żyta, Waldemar Marczewski

**Affiliations:** Plant Breeding and Acclimatization Institute, National Research Institute, Platanowa 19, 05-831 Młochów, Poland

**Keywords:** Potato cultivars, PVY, Hypersensitivity, Mapping, Marker-assisted selection

## Abstract

Potato virus Y (PVY) is one of the most important viruses affecting potato (*Solanum tuberosum*) production. In this study, a novel hypersensitive response (HR) gene, *Ny*-*2*, conferring resistance to PVY was mapped on potato chromosome XI in cultivar Romula. In cultivars Albatros and Sekwana, the *Ny*-*1* gene was mapped on chromosome IX. In cv. Romula, the local lesions appeared in leaves inoculated with the PVY^N-Wi^ isolate at 20 and 28 °C; PVY systemic infections were only occasionally observed at the higher temperature. In cvs. Albatros and Sekwana, expression of the necrotic reaction to virus infection was temperature-dependent. PVY^N-Wi^ was localized at 20 °C; at 28 °C, the systemic, symptomless infection was observed. We developed the B11.6_1600_ marker co-segregating with *Ny*-*2* and the S1d11 marker specific for the *Ny*-*1* gene. Fifty potato cultivars were tested with markers B11.6 and S1d11 and marker SC895 linked to the *Ny*-*1* gene in cv. Rywal. These results indicated the utility of these markers for marker-assisted selection of HR-like PVY resistance in potato breeding programs.

Potato virus Y (PVY) is the most harmful virus infecting potato crops (Valkonen 2007). In potato cultivars, *Ry* genes confer extreme resistance (ER) to all PVY strains. Plants expressing ER typically remain symptomless, with extremely low viral accumulation in inoculated leaves (Valkonen et al. 1996; Hämäläinen et al. 1998). Three genes that confer ER resistance were identified in potato: the *Ry*_*sto*_ gene (also designated as *Ry*-*f*_*sto*_) derived from *S. stoloniferum* on potato chromosome XII (Flis et al. 2005; Song et al. 2005); the *Ry*_*adg*_ gene from *S. tuberosum* ssp. *andigena* on chromosome XI (Hämäläinen et al. 1997), and *Ry*_*chc*_ from *S. chacoense* on chromosome IX (Sato et al. 2006). The hypersensitive response (HR) is another potato defense mechanism against PVY (Valkonen et al. 1996). The typical HR involves effective pathogen restriction in infected cells, which is associated with necrotic lesion generation at infection sites (Kang et al. 2005). Studies have shown that HR is responsible for limiting pathogen spread, which has the potential to result in systemic necrosis development (Valkonen et al. 1996). Rywal is the first potato cultivar in which temperature-dependent HR expression against PVY^O^ and PVY^N^ strains was detected. The *Ny*-*1* gene was mapped on potato chromosome IX (Szajko et al. 2008). Here we report mapping of HR-like genes conferring resistance to PVY in potato cultivars Albatros, Sekwana, and Romula, and the development of PCR markers useful for marker-assisted selection in potato breeding programs.

Three potato cultivars, Albatros, Romula and Sekwana, were evaluated for resistance to the isolate PVY^N-Wi^ (accession number Z70238). The PVY^N-Wi^ status was verified using the protocol reported by Lorenzen et al. (2006). Potato plants were grown for 2 weeks under greenhouse conditions, and transferred 1 week prior to infection experiments to growth chambers under controlled environmental conditions (20 or 28 °C, 16 h light at 100 mol/s/m^2^, 8 h dark). For each cultivar, six plants were mechanically inoculated with a sap extracted from the tobacco plants that were infected with PVY^N-Wi^. Inoculated potato plants were divided into two groups; one group was incubated at 20 °C, the other at 28 °C. Water-treated detached leaves and plants were used as negative controls. Samsun tobacco plants inoculated with PVY^N-Wi^ were used as positive controls. Hypersensitivity was visualized after 4–6 days. Nine days following inoculation, 1 g of inoculated and non-inoculated upper leaf samples from each of the plants was collected to detect the virus by RT-PCR, which was performed as described by Szajko et al. (2008) with minor modifications. In addition, ELISA tests were performed on inoculated and upper leaves 3 weeks post-inoculation at 20 °C. PVY monoclonal cocktail Bioreba AG kit (Reinach, Switzerland) was used. Experiments were repeated three times with similar results.

At 20 °C, cvs. Albatros and Sekwana exhibited a hypersensitive response to PVY^N-Wi^ infection in inoculated leaves. The virus was not detected in the upper, non-inoculated leaves. At 28 °C, systemic symptomless infections were observed. In cv. Romula, the local necrotic lesions appeared at 20 and 28 °C. Occasionally, HR failed to restrict PVY progression to upper, non-inoculated leaves of cv. Romula grown at 28 °C. Albatros (A) and Sekwana (Se) were crossed with the PVY-susceptible Dutch cultivar Accent (Ac) to obtain mapping populations A × Ac and Se × Ac with 42 and 58 F1 individuals, respectively. A segregating F1 population R × F was established consisting of 52 individuals derived from a cross between cv. Romula (R) and Polish cultivar Felka (F). Two tuber-derived plants per each F1 individual clone representing populations A × Ac, Se × Ac, and R × F were screened for HR to PVY^N-Wi^ infection at 20 °C. Plants which developed necrotic symptoms 6 days post-inoculation were classified as resistant. The following ratios of resistant to susceptible plants were detected: A × Ac − 24:18, Se × Ac − 30:28, and R × F − 24:28. The segregation ratios of 1:1 (χ^2^ = 0.85, *P* = 0.35; χ^2^ = 0.07, *P* = 0.79; and χ^2^ = 0.31, *P* = 0.58, respectively) confirmed the presence of single, dominant genes for HR to PVY in simplex states in all three resistant cultivars.

Five markers, S1d11, GP129, U38666, ShkB, and Nl27, were amplified in 20 μl of 20 mM Tris–HCl pH 8.4, 50 mM KCl, 1.5 mM MgCl_2_, 0.1 mM of each dNTP, 0.2 μM of each primer, 1 U Taq DNA polymerase (Invitrogen, Carlsbad, CA, USA), and 30 ng genomic DNA. MgCl_2_ concentration was increased to 3 mM for amplification of three markers: U276927, TG186, and TG591. In addition, 1 U of DreamTaq™ DNA polymerase and 1× buffer containing 2 mM MgCl_2_ (Fermentas, Vilnius, Lithuania) were used for marker B11.6. The following PCR parameters were employed: initial denaturation at 94 °C for 60 s followed by 40 cycles of denaturation at 93 °C for 25 s, annealing at 54 − 62 °C for 35 s (Table 1), and extension at 72 °C for 90 s, with a final extension at 72 °C for 5 min. The sequence characterized amplified region (SCAR) marker SC895 (NCBI GenBank accession EF555209) was amplified as described by Szajko et al. (2008). The At3g24050 (SGN U270244) marker was co-amplified with SC895 as an internal PCR control. At3g24050 primers comprised forward: 5′-CCTCTGGGGCCGAAAACACT-3′ and reverse: 5′-TCCATCACGAGCGAACACCAC-3′.

**Table 1:** CAPS markers used in genetic linkage maps

Chromosome no.	Marker name	Accession no.	Primer sequence (5′–3′)	*T*_a_ (°C)	Amplicon size (bp)	Restriction enzyme	Marker size (s) (bp)
IX	S1d11	AJ489115	f: GCCAAAAAGGGTAGGAAAAATG r: TCATCTTCACGAATCGGACTAAA	54	400	*Mnl*I	390
	TG591	SGN-M462	f: TCCAATCCGATGACCTCTG r: AGCTGCAAATCTACTCGTG	54	450	*Xap*I^a^	260
						*Hae*III^b^	310
	GP129	AJ487342	f: GTGGTAGCAAAGTATTCATC r: CGTTATCTGGACTCCTTTAG	54	500	*Xap*I	500
	U38666	SGN-U38666	f: AGCTGCCGTGTCCTGTATCA r: ACTCATGTTCACGCCACTTTCTTA	56	600	*Dde*I	550,600
	U276927	SGN-U276927	f: GCATTAGCGCAATTGGAATCCC r: GGAGAGCATTAGTACAGCGTC	54	1,250	*Dde*I	430
	TG186	SGN-M561	f: AACGGTGTACGAGATTTTAC r: ACCTACATAGATGAACCTCC	54	600	*Alu*I	490
XI	ShkB	M95201	f: CATCTTCTCCATAACCCTTTACC r: GCTCACAGTTCTCCACAAAATC	56	1,100	*Nla*III	1,000
	Nl27	AJ009720	f: CGGGAGTAGGCAAAACGACAATAGCAA r: ACTCAATCCTATATCAGCTCCAAAATCACAG	62	750	*Hin*fI	310
	B11.6	AC238172	f: ATAGCTTCAGGTCGGGAGATTTGT r: GATTGGGCTGCTGCTGTGG	60	1,800	*Eco*RI	1,600

*f* forward, *r* reverse

^a^Used for mapping of *Ny*-*1* in cv. Albatros

^b^Used for mapping of *Ny*-*1* in cv. Sekwana

Nine cleaved amplified polymorphic sequence (CAPS) markers were employed for mapping (Table 1). Four markers, TG591, S1d11, U38666, and TG186, exhibited utility in mapping the HR gene on potato chromosome IX in cv. Albatros. TG591 and S1d11, as well as GP129 and U276927, confirmed the presence of the HR gene in the same region of chromosome IX in cv. Sekwana (Fig. 1). The infection symptoms and the map positions of the PVY resistance loci in these cultivars were similar to those described for the *Ny*-*1* gene in cv. Rywal (Szajko et al. 2008). The HR genes in cvs. Albatros and Sekwana were therefore designated *Ny*-*1*. However, further molecular studies are necessary to determine whether *Ny*-*1* in cvs. Albatros, Rywal, and Sekwana reside at the same resistance locus, or whether these cultivars carry PVY resistance genes located at different loci on potato chromosome IX. Three DNA markers, Nl27, ShkB, and B11.6, were useful for mapping the HR-like gene *Ny*-*2* for PVY resistance on chromosome XI in cv. Romula (Fig. 1), in a resistance hotspot containing genes for qualitative and quantitative resistance to pathogens. The *Ry*_*adg*_ gene for ER to PVY has been previously mapped to this chromosome region (Hämäläinen et al. 1997). However, the diagnostic marker RYSC3 for the *Ry*_*adg*_ gene (Kasai et al. 2000) was not amplified in cv. Romula.

**Fig. 1:**
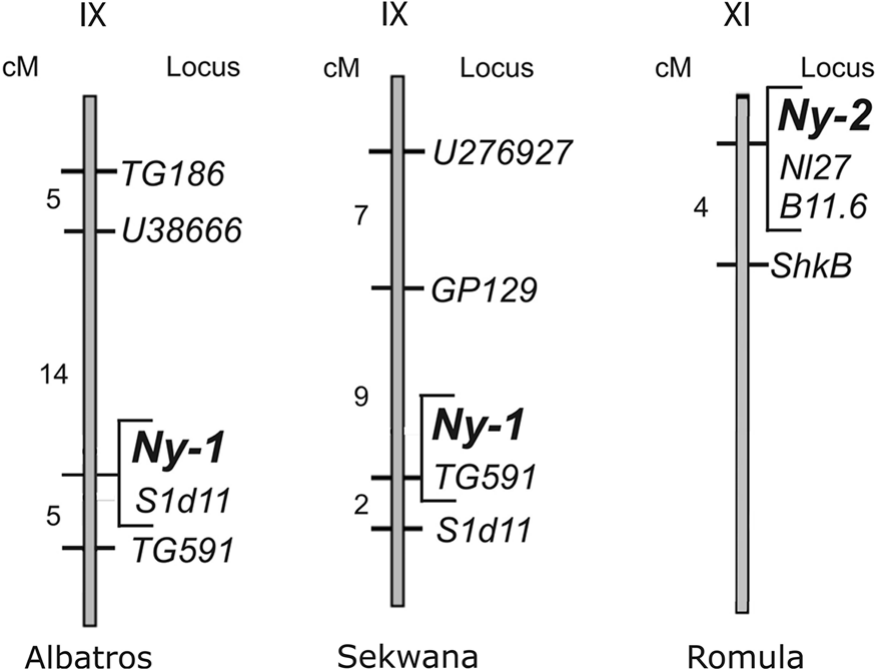
Positions of the loci *Ny*-*1* on chromosome IX in cvs. Albatros and Sekwana and *Ny*-*2* on chromosome XI cv. Romula. Genetic distances in cM are shown on the *left*. The map distances were calculated from recombination frequencies between DNA markers and resistance loci

Fifty potato cultivars (Table 2), bred in Poland, Germany, and The Netherlands, were tested for the presence of the SCAR marker SC895 and two CAPS markers, S1d11 and B11.6. Markers TG591 and Nl27 were not informative. Pedigree information (available for most cvs. at http://www.plantbreeding.wur.nl/potatopedigree/) shows that these cultivars represent various potato genetic backgrounds. They were classified into three groups: ER (extreme resistance to PVY), HR (hypersensitive), and S (susceptible). DNA of seven ER German cultivars (Assia, Barbara, Esta, Heidrun, Pirola, Ute, and Wega) were received from the Max Planck Institute for Plant Breeding Research, courtesy of Dr. C. Gebhardt. The remaining 43 were classified for PVY resistance at Młochów as previously described (Flis et al. 2005; Witek et al. 2006; Szajko et al. 2008; Valkonen et al. 2008). SC895, S1d11, and B11.6 were not detected in the sixteen cvs. susceptible to PVY and eighteen cvs. that expressed ER to PVY. In cvs. Mors and Syrena, which were also negative for all three markers, recombination events or the presence of another source of HR against PVY might be possible. SC895 was specific only to cv. Rywal. Therefore, this marker has potent utility for PVY resistance selection in progeny originated from the Rywal-derived source. The possibility that *Ny*-*1* and *Ny*-*2* genes are involved in resistance to PVY in the corresponding cvs. Koga, Neptun, Niagara, and Korona should be supported by additional genetic studies. Detection of the B11.6 marker in six cvs. from the ER group might be explained by epistatic expression of the *Ry* gene over *Ny* genes, where both types of PVY resistance genes are present in these cultivars, but the hypersensitive response is not induced. In diploid potato, this interaction type was exhibited between the PVY resistance genes *Ry*_*adg*_ and *Ny*_*adg*_ (Valkonen et al. 1994).

**Table 2:** Distribution of markers SC895, S1d11, and B11.6 in potato cultivars

Response to PVY	Potato cultivar	Presence of markers		
		SC895	S1d11	B11.6
HR	Rywal	+	−	−
	Albatros, Koga, Neptun, Niagara, Sekwana	−	+	−
	Korona, Romula	−	−	+
	Mors, Syrena	−	−	−
ER	Gabi, Jasia, Maryna, Santé, Ślęza, Sonda	−	−	+
	Ania, Assia, Baszta, Beata, Barbara, Danusia, Dunajec, Esta, Fregata, Heidrun, Hinga, Kuba, Nimfy, Pirola, Umiak, Ursus, Ute, Wega	−	−	−
S	Accent, Balbina, Delikat, Drop, Felka, Grot, Karatop, Karlena, Oda, Orłan, Pirol, Tara, Tokaj, Triada, Wawrzyn, Wolfram	−	−	−

*HR* hypersensitive response, *ER* extreme resistance, *S* susceptible, + presence of marker; − absence of marker

In cultivated potato, a cross between a clone possessing two resistance alleles and a susceptible clone, or between two simplex resistant parents, resulted in a large percentage of resistant clones in F1 generations (Solomon-Blackburn and Mackay 1993). The PCR-based markers developed in this study might facilitate the production of PVY-resistant tetraploid potato clones with double HR gene doses, or the combination of HR and ER resistance factors, thereby improving breeding efficiency for PVY resistance in cultivated potato.
